# Common prosperity level evaluation: A comprehensive method based on probabilistic linguistic ordered weighted distance measure, prospect theory, and TOPSIS

**DOI:** 10.3389/fpsyg.2023.1152333

**Published:** 2023-03-15

**Authors:** Erhua Zhang, Feifan Yu, Ting Jiang, Shouzhen Zeng, Dandan Wang

**Affiliations:** ^1^School of Business, Ningbo University, Ningbo, China; ^2^School of Statistics and Mathematics, Zhejiang Gongshang University, Hangzhou, China; ^3^College of Economics and Management, Nanjing University of Aeronautics and Astronautics, Nanjing, China

**Keywords:** common prosperity, probabilistic linguistic term, prospect theory, ordered weighted distance measure, TOPSIS

## Abstract

**Introduction:**

Common prosperity is a major research project in China, and the scientific measurement and evaluation of common prosperity is very important.

**Methods:**

In this study, firstly, we construct a comprehensive evaluation index system for the common prosperity level (CPL). We then develop an evaluation model of CPL based on prospect theory, probabilistic linguistic ordered weighted distance measure, and the TOPSIS method, wherein we use a probabilistic linguistic term set (PLTS) to describe the uncertainty and complexity of the assessment process. Above all, we use prospect theory to reflect the preferences of experts to meet the unique needs for the evaluation of common prosperity. Moreover, we apply the proposed evaluation index system and model to evaluate the CPL of Zhejiang Province, China's first common prosperity demonstration zone, as an example to conduct relevant research. The advantages and effectiveness of the proposed method are verified by the sensitivity and comparative analysis.

**Results:**

The findings prove that the application of the new PLTS evaluation framework in CPL assessment is robust.

**Discussion:**

We propose specific suggestions for improving the development of common prosperity.

## 1. Introduction

The idea of common prosperity in China can be traced back to the Spring and Autumn and the Warring States Periods, approximately 2,000 years ago. Originally, it represented absolute egalitarianism. However, in contemporary China, the purpose of common prosperity is to promote fair income distribution and improve people's living standards, which also includes ideas such as sharing and development (Chen et al., 2022). In the existing research, some scholars highlight that social progress should simultaneously examine the overall development level and the living conditions of people with low standards of living (Kakwani et al., 2022; Zhang J. et al., 2022). Therefore, common prosperity is the achievement of a well-fed standard of living for all members of society. It is a differential wealth based on universal wealth but not egalitarianism. Common prosperity is the integration of two aspects—common and prosperity. The latter can be expressed as the abundance of social wealth and advanced productivity. Common prosperity means that members of society achieve a good standard of living through labor, thereby eliminating the polarization between the rich and the poor (Qian et al., 2021).

Common prosperity is an important requirement of socialism and an essential feature of Chinese-style modernization (Ja, 2022). The practice of common prosperity in China spans two centuries. As early as the 1950s, it is frequently mentioned by the Chinese government. The implementation of reform and opening up and the slogan first wealth to drive wealth later indicate that the construction of common prosperity has entered a new stage (Kong, 2022). In recent years, in the context of building a moderately prosperous society in China, the government is vigorously promoting the ideal of achieving common prosperity. In 2021, Zhejiang province was designated a common prosperity demonstration zone, shouldering the important historical mission of developing and building a high-quality and common prosperity area.

However, exploring the implementation and development of the common prosperity level (CPL) is more complicated than addressing other practical problems. In the context of modern research problems of increasing depth, there are obvious restrictions on traditional evaluation methods based on accurate values. The main reason is that the accurate evaluation information makes it difficult to describe policymakers' preferences for indicators and programs and also does not reveal the uncertainty of prospects. In the face of relatively complex evaluation objects, evaluators or decision-makers may be hesitant to evaluate, which leads to the lack of a certain basis for the results. Therefore, the traditional evaluation methods based on accurate values have obvious shortcomings in dealing with complex problems; that is, the data in the evaluation process cannot be completely and accurately obtained in practical cases. Therefore, we adopt the fuzzy evaluation method in which experts describe their evaluations and preferences through various fuzzy assessments in a complex multi-index comprehensive evaluation environment.

The fuzzy set theory (Zadeh, 1965) was first invented by Zadeh in 1965. Later, fuzzy evaluation branch methods evolved, including using language variables (Zadeh, 1974), which is one of the more understandable methods to represent fuzzy concepts. To solve the problem of choosing among multiple linguistic terms, Rodriguez combined the linguistic term set (Rodriguez et al., 2012) and the hesitant fuzzy set (Torra, 2010) and proposed a hesitant fuzzy linguistic term set (HFLTS) that includes multiple linguistic variables of the same weight. However, due to the limitations of the weights of linguistic terms, HFLTSs lack applicability in practical problems that need to reflect different degrees of importance. Therefore, considering the diversity and flexibility of linguistic information expression, Pang et al. (2016) proposed a probability linguistic term set (PLTS) by introducing probability information based on the HFLTS. PLTS has been widely used for its special advantages, such as project evaluation (Peng et al., 2020; Shen et al., 2020), environmental impact assessment (Tian et al., 2020; Su et al., 2023), and text sentiment analysis (Song et al., 2020; Yu and Pan, 2021).

Considering the great fuzziness of the concept of common prosperity, there exist difficulties in determining the weight of fuzzy indicators when using fuzzy evaluation to solve the problem of common prosperity. While prospect theory (Kahneman and Tversky, 1979) has a good performance in dealing with such problems, which is specifically embodied in that it can reflect the different psychological characteristics of decision-makers when they face gains and losses in actual decisions. Prospect theory can help people make decisions under uncertain circumstances and can reflect the preferences of decision-makers. Hence, prospect theory has attracted extensive attention and has been gradually applied in various areas, so it is introduced in the process of CPL evaluation and the fuzzy set theory in this study.

Similar to other evaluation problems (Zeng et al., 2013a, 2017), using the classic distance between PLTS as the basis for the evaluation and measurement of CPL problems is inadequate. Therefore, we need to explore a suitable distance measure to handle the CPL problems. There exist various distance metrics to choose from when the decision-maker makes a decision, such as the Minkowski distance (Merigó and Gil-Lafuente, 2008), Hamming distance (Hamming, 1950), and ordered weighted distance (OWD) (Xu and Chen, 2008; Merigo and Casanovas, 2011). Among these, the OWD, a useful extension of the ordered weighted operator (OWA) (Yager, 1988), has the advantage of obtaining better results and reaching a consensus faster. Then, the weight of the OWD measure can be set according to the requirements of the problem to enhance or alleviate the influence of large or small differences in the integrated results. In addition, Xu and Xia (2011) combined the OWD method with a hesitant fuzzy case and extended the theory of hesitant fuzzy OWD. Zeng et al. (2013a) extended the OWD to an intuitionistic fuzzy environment and proposed the intuitionistic fuzzy OWD measure. Recently, Liu et al. (2022) proposed a combination of PLTS and OWD and developed the probabilistic linguistic term OWD (PTLOWD) measure enriching distance theory in the context of PLTSs. Additional extensions and research concerning the OWD measure are available (Zeng et al., 2012, 2013b, 2017, 2023; Peng et al., 2014; Dai, 2020; Yang et al., 2022).

Based on the above literature review, we find that there are still few studies on the application of the prospect theory in the direction of PLTOWD. In this study, the prospect theory endows the PLTOWD operator with the ability to consider the influence of psychological factors on experts in decision-making procedures, meaning that it can overcome the subjective value cognition caused by decision anticipation to reflect the preference of decision-makers.

The gap between rural and urban areas has been narrowing, and the residents' happiness index has steadily increased, indicating that the common prosperity of Zhejiang province has begun to materialize. However, we identified the following several issues to be addressed as follows:

1. The CPL evaluation process is ambiguous and complex, and a scientific evaluation index system for the level of common prosperity is required.
2. The improvement of the overall prosperity of Zhejiang province does not mean that all prefecture-level cities have reached this level, and there is a lack of an effective method for common prosperity evaluation in complex environments.
3. The existing PLTOWD has the disadvantage of not considering the preferences of the decision-makers.

Therefore, we select the prospect theory to solve the current drawback in this study. Furthermore, we apply the PTLTOWD measure to the TOPSIS model to evaluate the common prosperity level of cities in Zhejiang province. We derive an evaluation of construction experience and institutional models, leading to scientific and objective conclusions. The contributions of this study are reflected in the following four aspects:

1. We build a set of scientific evaluation index systems for the development of common prosperity;
2. We propose a new PLTOWD measure based on prospect theory;
3. We present a new evaluation framework based on the proposed PTPLTOWD measure to TOPSIS;
4. We extend the application of the presented method to the field of common prosperity evaluation and use it to evaluate the common prosperity level of cities in Zhejiang province, obtaining a scientific evaluation result.

The rest structure of this manuscript is organized as follows: Section 2 presents a basic knowledge review. In section 3, the comprehensive evaluation index system for CPL is introduced. Section 4 offers a new evaluation framework based on PTPLTOWD and TOPSIS tool. Section 5 provides an empirical test on the common prosperity practice in Zhejiang province in China. Section 6 offers conclusions, main limitations, and future directions.

## 2. Preliminaries

We introduce some basic concepts of PLTSs and then outline the concepts of PLTOWD and prospect theory in this section.

### 2.1. Probabilistic linguistic term

**Definition 1** (Pang et al., 2016): Let *S* = {*s*_α_ |α = 0, 1, 2, ⋯ , τ } be a language term set (LTS); then, a probabilistic linguistic term set (PLTS) is defined as follows:


(1) $$\begin{array}{l} L\left(p\right)=\{L^{\left(k\right)}\left(p^{\left(k\right)}\right)|L^{\left(k\right)}\;\in S,p^{\left(k\right)}\geq 0,k\\ \;=1,2,\cdots \;,\#L\left(p\right),\sum_{k=1}^{\#L\left(p\right)}p^{\left(k\right)}\leq 1\}, \end{array}$$

where *L*^(*k*)^ (*p*^(*k*)^) denotes the language term *L*^(*k*)^ with probability information *p*^(*k*)^ and *#L*(*p*) is the number of different language terms in set *L*(*p* ).

**Definition 2** (Pang et al., 2016): Given a PLTS (*p*), satisfying the condition: $\sum_{k=1}^{\#L\left(p\right)}p^{\left(k\right)}<1$, then its associated PLTS


(2) $$\begin{array}{l} \hat{L}\left(p\right)=\left\{L^{\left(k\right)}\left({\hat{p}}^{\left(k\right)}\right)|k=1,2,\cdots \;,\#L\left(p\right)\;\right\} \end{array}$$

is called the standardized set of PLTSs, where ${\hat{p}}^{\left(k\right)}=p^{\left(k\right)}/\sum_{k=1}^{\#L\left(p\right)}p^{\left(k\right)},k=1,2,\cdots \;\#L\left(p\;\right)$.

**Definition 3** (Pang et al., 2016): Suppose *L*_1_ (*p*) and *L*_2_ (*p*) are two different PLTSs denoted as $L_{1}\left(p\right)=\left\{L_{1}^{\left(k\right)}\left(p_{1}^{\left(k\right)}\right)|k=1,2,\cdots \;,\#L_{1}\left(p\right)\;\right\}$ and $L_{2}\left(p\right)=\left\{L_{2}^{\left(k\right)}\left(p_{2}^{\left(k\right)}\right)|k=1,2,\cdots \;,\#L_{2}\left(p\right)\;\right\}$, respectively. *#L*_1_ (*p*) and *#L*_2_ (*p*) represent the number of language terms in *L*_1_ (*p*) and *L*_2_ (*p*), respectively. For *#L*_1_ (*p*) > *#L*_2_ (*p*), we add *#L*_1_ (*p*)−*#L*_2_ (*p*) terms to *L*_2_ (*p*), making the number of language terms in *L*_1_ (*p*) and *L*_2_ (*p*). The newly added language term is the smallest in *L*_2_ (*p*), and its probability is 0.

**Definition 4** (Pang et al., 2016): Let *L*(*p*) = {*L*^(*k*)^ (*p*^(*k*)^)|*k* = 1, 2, ⋯ *#L*(*p*) } be a PLTS and *r*^(*k*)^ be the subscript of the language term *L*^(*k*)^. Then, the score function of *L* (*p*) is defined as follows:


(3) $$\begin{array}{l} E\left(L\left(p\right)\right)=s_{\overset{¯}{\alpha }}. \end{array}$$

Among them, $\overset{¯}{\alpha }=\sum_{k=1}^{\#L\left(p\right)}\left(r^{\left(k\right)}p^{\left(k\right)}\right)/\sum_{k=1}^{\#L\left(p\right)}p^{\left(k\right)}$.

**Definition 5** (Pang et al., 2016): Let *L*(*p*) = {*L*^(*k*)^ (*p*^(*k*)^)|*k* = 1, 2, ⋯ *#L*(*p*)} be a PLTS and *r*^(*k*)^ be the subscript of the language terms *L*^(*k*)^, $E\left(L\left(p\right)\right)=s_{\overset{¯}{\alpha }}$, and $\overset{¯}{\alpha }=\sum_{k=1}^{\#L\left(p\right)}\left(r^{\left(k\right)}p^{\left(k\right)}\right)/\sum_{k=1}^{\#L\left(p\right)}p^{\left(k\right)}$. Then, we can define the deviation function *L* (*p*) as follows:


(4) $$\begin{array}{l} \sigma \left(L\left(p\right)\right)={\left(\overset{\#L\left(p\right)}{\underset{k=1}{\sum }}{\left(p^{\left(k\right)}\left(r^{\left(k\right)}-\overset{¯}{\alpha }\right)\right)}^{2}\right)}^{1/2}/\overset{\#L\left(p\right)}{\underset{k=1}{\sum }}p^{\left(k\right)} \end{array}$$

**Definition 6** (Pang et al., 2016): Let *L*_*i*_ (*p*) (*i* = 1, 2) be any two PLTSs, then

1. If *E* (*L*_1_ (*p*)) > *E* (*L*_2_ (*p*)), then *L*_1_ (*p*) ≻ *L*_2_ (*p*) ;
2. If *E* (*L*_1_ (*p*)) = *E* (*L*_2_ (*p*)), then
  - If σ (*L*_1_ (*p*)) > σ(*L*_2_ (*p*)), then *L*_1_ (*p*) ≺ *L*_2_ (*p* );
  - If σ (*L*_1_ (*p*)) = σ(*L*_2_ (*p*)), then *L*_1_ (*p*) ~ *L*_2_ (*p* ).

**Definition 7** (Pang et al., 2016): Assuming that given a set of probabilistic linguistic terms $L_{i}\left(p\right)=\left\{{L_{i}}^{\left(k\right)}\left({p_{i}}^{\left(k\right)}\right)|k=1,2,\cdots \;\#L_{i}\left(p\right)\;\right\}\left(i=1,2\right)$, where *#L*_1_ = *#L*_2_, the Hamming distance of *L*_1_ (*p*) and *L*_2_ (*p*) is as follows:


(5) $$\begin{array}{l} d_{\omega }\;(L_{1},L_{2})=\frac{1}{\#L_{1}}\overset{\#L_{1}}{\underset{l=1}{\sum }}\left|p_{1}^{\left(l\right)}\Delta \left(L_{1}^{\left(l\right)}\right)-p_{2}^{\left(l\right)}\Delta \left(L_{2}^{\left(l\right)}\right)\right|. \end{array}$$

Given a probabilistic linguistic vector *L*_*i*_ = (*L*_*i*1_ (*p*), *L*_*i*2_ (*p*), ⋯ , *L*_*in*_ (*p*)) (*i* = 1, 2), the weighted Hamming distance of *L*_1_ and *L*_2_ is as follows:


(6) $$\begin{array}{l} d_{\omega }\;(L_{1},L_{2})=\overset{n}{\underset{j=1}{\sum }}\omega_{j}d\;(L_{1j}\;(p),L_{2j}\;(p)), \end{array}$$

where *d* (*L*_1*j*_ (*p*), *L*_2*j*_ (*p*)) is the Hamming distance of *L*_1*j*_ (*p*) and *L*_2*j*_ (*p*), and ω_*j*_ is the corresponding weight that satisfies ω_*j*_ ∈ [0, 1] and $\sum_{j=1}^{n}\omega_{j}=\;1$.

### 2.2. PLTOWD

The current method for the probabilistic linguistic distance measurement is complicated and tedious. In this context, the PLTOWD operator is introduced (Liu et al., 2022). PLTOWD can simplify the operation between elements of probabilistic linguistic terms, thereby improving operational efficiency. Assuming that *L*_1_ (*p*) and *L*_2_ (*p*) are two PLTSs, then


(7) $$\begin{array}{l} PLTOWD\;(a,b)={\left(\overset{n}{\underset{j=1}{\sum }}\omega_{j}{\left(d_{PLT}\;(a_{\sigma \left(j\right)},b_{\sigma \left(j\right)})\right)}^{\lambda }\right)}^{\frac{1}{\lambda }} \end{array}$$

is the ordered weighted distance of the PLTSs *a* and *b*, where $\omega ={\left(\omega_{1},\omega_{2},\cdots \;,\omega_{n}\right)}^{T}$ is the weight vector associated with the PLTOWD measure satisfying ω_*j*_ ∈ [0, 1] and $\sum_{j=1}^{n}\omega_{j}=1$. The subscript (σ (1), σ (2), ⋯ , σ (*n*)) is a permutation of (1, 2, ⋯ , *n*) such that *d* (*a*_σ(*j*−1)_, *b*_σ(*j*−1)_) ≥ *d* (*a*_σ(*j*)_, *b*_σ(*j*)_), where *d*_*PLT*_ (*a*_*j*_, *b*_*j*_) is the distance between the probabilistic linguistic terms *a*_*j*_ and *b*_*j*_.

### 2.3. Prospect theory

Prospect theory describes the psychological changes of decision-makers when gains or losses are contemplated, thereby showing changes in subjective value perceptions and reflecting the preferences of decision-makers. The specific form of the prospect theory value function is defined as follows (Kahneman and Tversky, 1979):


(8) $$\begin{array}{l} v\;(\Delta x)=\{\begin{array}{c} {\left(\Delta x\right)}^{\alpha },\;\Delta x\geq 0,\\ -\theta \;{\left(-\Delta x\right)}^{\beta },\;\Delta x<0,\; \end{array} \end{array}$$

where Δ*x* is the size of the deviation of *x* from a certain reference point *x*_0_; Δx ≥ 0 denotes the gain; and Δx < 0 represents the loss. α, β reflects the sensitivity of decision-makers to gains and losses, while θ is the loss aversion coefficient. According to the analysis and research of Tversky and Kahneman (Tversky and Kahneman, 1992), α = β = 0.88 *and θ* = 2.25 iare congruent with human decision-making psychology.

## 3. Comprehensive evaluation index system for CPL

**Figure 1:**
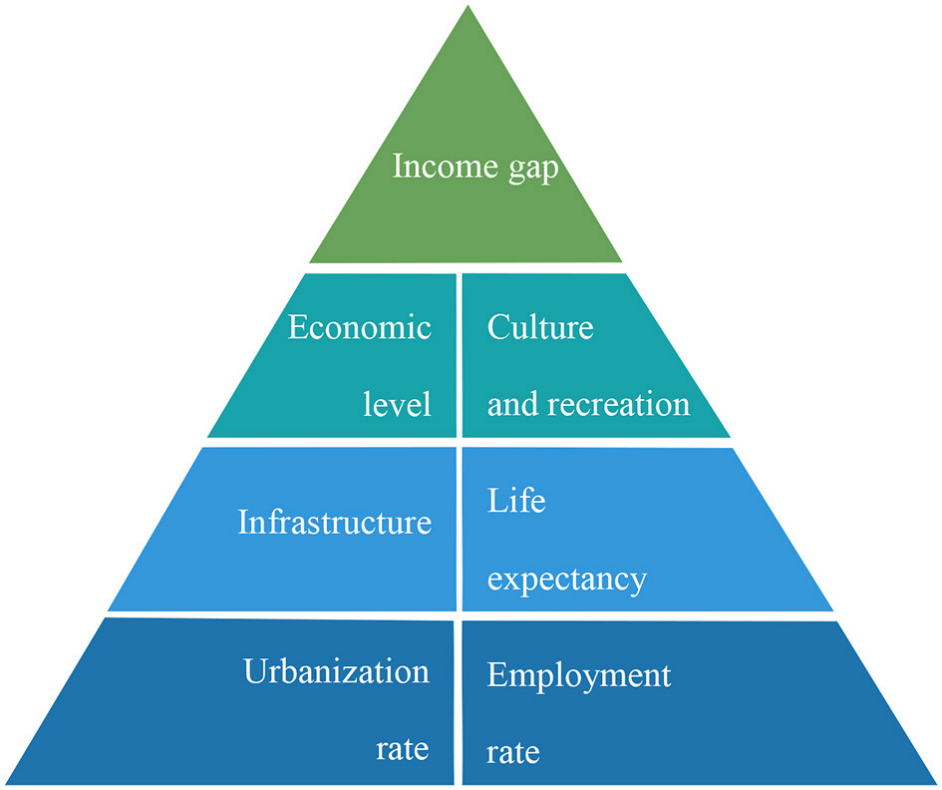
Evaluation system of CPL.

Based on current research about CPL, we construct a novel evaluation index system including seven aspects (Figure 1) that can best reflect the development of common prosperity, namely, income gap, economic level, culture and recreation, infrastructure, urbanization rate, life expectancy, and employment rate in this section.

### 3.1. Income gap

Common prosperity means “rich for all,” which represents the common prosperity of all the people, not the prosperity of some regions or groups. To achieve common prosperity, it is necessary to deal with the problem of unbalanced and inadequate development, and the outstanding manifestation of this problem is the income gap among citizens. In addition, the income gap significantly reflects the degree of sharing of the development fruits of common prosperity. It is an important indicator to measure the implementation effect of common prosperity (Zhao and Jiao, 2022).

Economic level: Common prosperity is based on a higher level of social productive forces, and the emancipation and development of productive forces can build a material foundation for the realization of common prosperity, which is also a prerequisite. Therefore, it is in line with our research to choose the overall economic level of society as an important evaluation index (Li et al., 2022).

### 3.2. Culture and recreation

While considering the material standard of living, we should also think about whether the spiritual life of citizens is rich. For example, whether the various activities, cultural exchanges, and socializing organized by the government can meet the recreational needs of citizens in their spare time (Wang et al., 2022). This is also an important dimension of the evaluation of common prosperity.

### 3.3. Infrastructure

Infrastructure denotes the material engineering facilities that provide public services for social production and people's life, including water and electricity, transportation, medical care, education, and other general material conditions to ensure social survival and economic development. In modern society, the more economic and social development, the higher the demand for infrastructure; a sound infrastructure also plays a big role in achieving common prosperity and improving citizens' satisfaction (Tang et al., 2022).

### 3.4. Urbanization rate

The urbanization rate is a measure of the urbanization level. From the perspective of equalization of factor remuneration, with the increase of urbanization rate, the income gap between rural and urban areas will gradually narrow in the process of transferring rural labor to cities; that is, the development of urbanization will do well to narrow the income gap between rural and urban areas, thus promoting the realization of common prosperity. Therefore, the urbanization rate can be regarded as an important variable in measuring common prosperity (Guan et al., 2022).

### 3.5. Life expectancy

Economic conditions and the level of healthcare limit people's longevity. The average life expectancy is a key indicator to measure the health level of the residents of a region, a country, or a nation. It can clearly reflect the quality of life of a society and is also an important embodiment of common prosperity (Pu et al., 2022).

### 3.5. Employment rate

A high employment rate indicates that people live and work in peace and contentment, which is important for increasing incomes and promoting social stability. Since the epidemic, the proportion of flexible employment in China has been on the rise, improving the employment rate and the quality of employment and contributing to the expansion of the middle-income group and the realization of common prosperity (Meng et al., 2022).

## 4. An evaluation framework based on PTPLTOWD and TOPSIS method

### 4.1. PTPLTOWD measure

The PLTOWD measure is an effective tool for processing qualitative information and its corresponding probability or importance. Although the PLTOWD expands the distance measure of probabilistic language, it is still too rigid in algorithmic composition and thinking, ignoring the psychological conditions and preferences of decision-makers, making the overall decision flexibility limited. In view of the above considerations, we have combined PLTOWD with the prospect theory, which has clear advantages: (1) It can incorporate the psychological factors of experts in the decision-making process; (2) it accounts for the expected gains and losses of the decision generating subjective value perceptions.

In this section, combined with the prospect theory, we proposed the prospect theory PLTOWD (PTPLTOWD) operator enriching the distance theory under the condition of probabilistic linguistics and applying it to the TOPSIS method. Let *A* = *a*_1_, *a*_2_, ⋯ , *a*_*m*_ be the scheme set, *C* = *c*_1_, *c*_2_, ⋯ , *c*_*n*_ be the attribute set, and construct a decision matrix = [*P*_*ij*_]_*m*×*n*_. The main steps of construction of the PTPLTOWD are presented below:

**Step 1:** Selection of reference points. When applying the prospect theory, the choice of reference points directly affects the calculation of the value function. In practice, decision-makers have different subjective preferences, thus determining different reference points.

**Assumption 1**: For optimistic decision-makers, when the distance measure is cost type, the decision reference point is $D^{L}=d_{PLT}\;(a_{\sigma (j)},b_{\sigma (j)}),j=1,2,\cdots \;n$ ; when the distance measure is of the benefit type, then the decision reference point is $D^{L}=D^{L}=d_{PLT}\;(a_{\sigma (j)},b_{\sigma (j)}\;).$

**Assumption 2:** For pessimistic decision-makers, when the distance measure is cost type, the decision reference point is $D^{B}=d_{PLT}\;(a_{\sigma (j)},b_{\sigma (j)}),j=1,2,\cdots \;n$; when the distance measure is of benefit type, then the decision reference point is $D^{B}=d_{PLT}\;(a_{\sigma (j)},b_{\sigma (j)}\;).$

**Assumption 3:** For neutral decision-makers, whether the distance measure is cost or benefit, the decision reference point is $D^{Z}=\frac{1}{n}\sum_{j=1}^{n}d_{PLT}\left(a_{\sigma \left(j\right)},b_{\sigma \left(j\right)}\;\right).$

In this study, we suppose the decision-makers are neutral and then construct a PTPLTOWD based on prospect theory and PLTOWD.

**Step 2:** Calculate the distance from each scheme to the reference point. In this step, we need to calculate the Δ*x* in Equation (8), which represents the distance of each scheme from the reference point in prospect theory.

For optimistic decision-makers of assumption 1:


$$\begin{array}{l} \Delta x=d\;(a_{i},D^{L})=\left\{\left(P_{i1},D_{1}^{L}\right),\left(P_{i2},D_{2}^{L}\right),\cdots \;,\left(P_{im},D_{m}^{L}\right)\right\} \end{array}$$

For pessimistic decision-makers of assumption 2:


$$\begin{array}{l} \Delta x=d\;(a_{i},D^{B})=\left\{\left(P_{i1},D_{1}^{B}\right),\left(P_{i2},D_{2}^{B}\right),\cdots \;,\left(P_{im},D_{m}^{B}\right)\right\} \end{array}$$

For neutral decision-makers of assumption 3:


$$\begin{array}{l} \Delta x=d\;(a_{i},D^{Z})=\left\{\left(P_{i1},D_{1}^{z}\right),\left(P_{i2},D_{2}^{z}\right),\cdots \;,\left(P_{im},D_{m}^{z}\;\right)\right\}. \end{array}$$

**Step 3:** Acquire the prospect value and weight. The calculation process of the prospect value is as follows: With *D*^*Z*^ as the reference point, Equation (8) is used to calculate the prospect value function of each scheme. For *V*_*j*_ can be positive or negative, and distance measurement can be divided into cost and benefit types. To make the prospect value all positive, the following conversion rules are implemented for all prospect values:


(9) $$\begin{array}{l} V_{j}^{\prime }=max\;\left\{V_{1},V_{2},\cdots \;,V_{n}\right\}-min\;\left\{V_{1},V_{2},\cdots \;,V_{n}\right\}+V_{j}, \end{array}$$

(10) $$\begin{array}{l} V_{j}^{\prime \prime }=max\;\left\{V_{1},V_{2},\cdots \;,V_{n}\right\}-min\;\{V_{1},V_{2},\cdots \;,V_{n}\}-V_{j}, \end{array}$$

The above Equation (9) represents the conversion of the prospect value calculated in the case of benefit-based distance measurement, and Equation (10) represents the conversion of the foreground value calculated in the case of cost-based distance measurement. After the conversion, $V_{j}^{\prime }$ and $V_{j}^{\prime \prime }$ are both positive numbers, and the higher the value, the better.

For convenience, we utilize *V*_*j*_ to represent $V_{j}^{\prime }$ and $V_{j}^{\prime \prime }$. Furthermore, in the case application, all indicators are converted uniformly into benefit-based distance measurement in advance; that is, the prospect value *V*_*j*_ will be transformed according to Equation (9). The weight calculation method associated with the ordered weighted distance measure based on prospect theory is shown as follows:


(11) $$\begin{array}{l} \varphi_{j}=\frac{V_{j}}{\sum_{j=1}^{n}V_{j}}. \end{array}$$

After the foreground value calculated in different situations is converted, it is normalized to satisfy φ_*j*_ ∈ [0, 1] and $\sum_{j=1}^{n}\varphi_{j}=\;1.$

**Step 4:** Determine the index weight. In this study, we construct a multi-objective programming model to minimize $d_{\omega }\;\left(P_{ij},P_{{}^{*}j}\right)$ and maximize *d*_ω_ (*P*_*ij*_, *P*_*#j*_) to acquire the weight of attributes:


$$\begin{array}{l} \{\begin{array}{c} \min d_{\omega }\;(P_{ij},P_{{}^{*}j})\;\left(i=1,2,\cdots \;,m\right)\\ \max d_{\omega }\;(P_{ij},P_{\#j})\;\left(i=1,2,\cdots \;,m\right)\\ s.t.\{\begin{array}{c} \sum_{j=1}^{n}{\left(\omega_{j}\right)}^{2}=1\\ \omega_{j}\geq 0,\;j=1,2,\cdots \;,n \end{array} \end{array} \end{array}$$

Furthermore, the above multi-objective programming model can be converted into


$$\begin{array}{l} \{\begin{array}{c} \min\sum_{j=1}^{n}\omega_{j}\sum_{i=1}^{m}\left[d_{\omega }\;(P_{ij},P_{{}^{*}j})-d_{\omega }\;(P_{ij},P_{\#j})\right]\\ s.t.\{\begin{array}{c} \sum_{j=1}^{n}{\left(\omega_{j}\right)}^{2}=1\\ \omega_{j}\geq 0,\;j=1,2,\cdots \;,n \end{array} \end{array} \end{array}$$

Let $x_{j}=\sum_{i=1}^{m}\left[d_{\omega }\left(P_{ij},P_{{}^{*}j}\right)-d_{\omega }\left(P_{ij},P_{\#j}\right)\right]$, then we construct the Lagrange function $F=\sum_{j=1}^{n}\omega_{j}x_{j}+\lambda \left[\sum_{j=1}^{n}{\left(\omega_{j}\right)}^{2}-1\right]$, where λ is the Lagrange multiplier. When the partial derivative of *F* with respect to ω_*j*_ (*j* = 1, ⋯ , *n*) is 0, we have:


$$\begin{array}{l} \{\begin{array}{l} \frac{\partial F}{\partial \omega_{j}}=x_{j}+2\lambda \omega_{j}=0\\ \;\frac{\partial F}{\partial \lambda }={\left(\omega_{j}\right)}^{2}-1=0 \end{array} \end{array}$$

After solving the above equation, the optimal solution can be obtained as follows:


$$\begin{array}{l} \omega_{j}^{{}^{*}}=\frac{x_{j}}{\sqrt{\sum_{j=1}^{n}{\left(x_{j}\right)}^{2}}}\;,j=\;1,2,\cdots \;,n, \end{array}$$

Considering the normalization constraint of attribute weight, the final attribute weight can be obtained by referring to the following mathematical expression:


(12) $$\begin{array}{l} \omega_{j}=\frac{\{\sum_{i=1}^{m}{\left[d\;(P_{ij},P_{{}^{*}j})-d\;(P_{ij},P_{\#j})\right]}^{2}}{\sum_{j=1}^{n}{\left\{\sum_{i=1}^{m}\left[d\;(P_{ij},P_{{}^{*}j})-d\;(P_{ij},P_{\#j})\right]\right\}}^{2}}. \end{array}$$

**Step 5:** Construct the PTPLTOWD measure. Combining the PLTOWD measure [Equation (7)] with the weights obtained from the prospect theory [Equation (11)], we have


(13) $$\begin{array}{l} PTPLTOWD\;(a,b)={\left(\overset{n}{\underset{j=1}{\sum }}\varphi_{j}{\;(\omega_{j}d_{PLT}\;(a_{\sigma \left(j\right)},b_{\sigma \left(j\right)}))}^{\lambda }\right)}^{\frac{1}{\lambda }}, \end{array}$$

where, φ_*j*_ is the weight obtained from prospect theory as well as the OWD position weight, ω_*j*_ is the index weight.

### 4.2. A PTPLTOWD-TOPSIS evaluation framework

The PTPLTOWD-TOPSIS evaluation framework is mainly composed of introducing the TOPSIS method into the PTPLTOWD measure, not only achieving the orderly aggregation of diverse information but also reflecting the subjective value perception of decision-makers.

Suppose a multi-attribute decision problem in a PLT environment contains *m* alternatives and *n* decision attributes. Let *A* = *A*_1_, *A*_2_, ⋯ , *A*_*m*_ be the scheme set and *C* = *c*_1_, *c*_2_, ⋯ , *c*_*n*_ be the attribute set. Let the attribute weight vector be $\omega ={\left(\omega_{1},\omega_{2},\cdots \;,\omega_{n}\right)}^{T}$, satisfying ω ∈ [0, 1] and ${\sum_{i=1}^{n}\omega }_{i}=1$. Using *S* = {*s*_α_|α = 0, 1, ⋯ , τ}, the decision-maker provides the evaluation value of the alternatives in the form of PLT values under the evaluation attribute and thus constructs the decision matrix *R* = [*P*_*ij*_]_*m*×*n*_ expressed as follows:


$$\begin{array}{l} R={\left[P_{ij}\right]}_{m\times n}=\left[\begin{array}{cccc} P_{11}\; & P_{12}\; & \cdots \; & P_{1n}\\ P_{21}\; & P_{22}\; & \cdots \; & P_{2n}\\ \vdots \; & \vdots \; & \ddots \; & \vdots \\ P_{m1}\; & P_{m2}\; & \cdots \; & P_{mn} \end{array}\right], \end{array}$$

**Figure 2:**
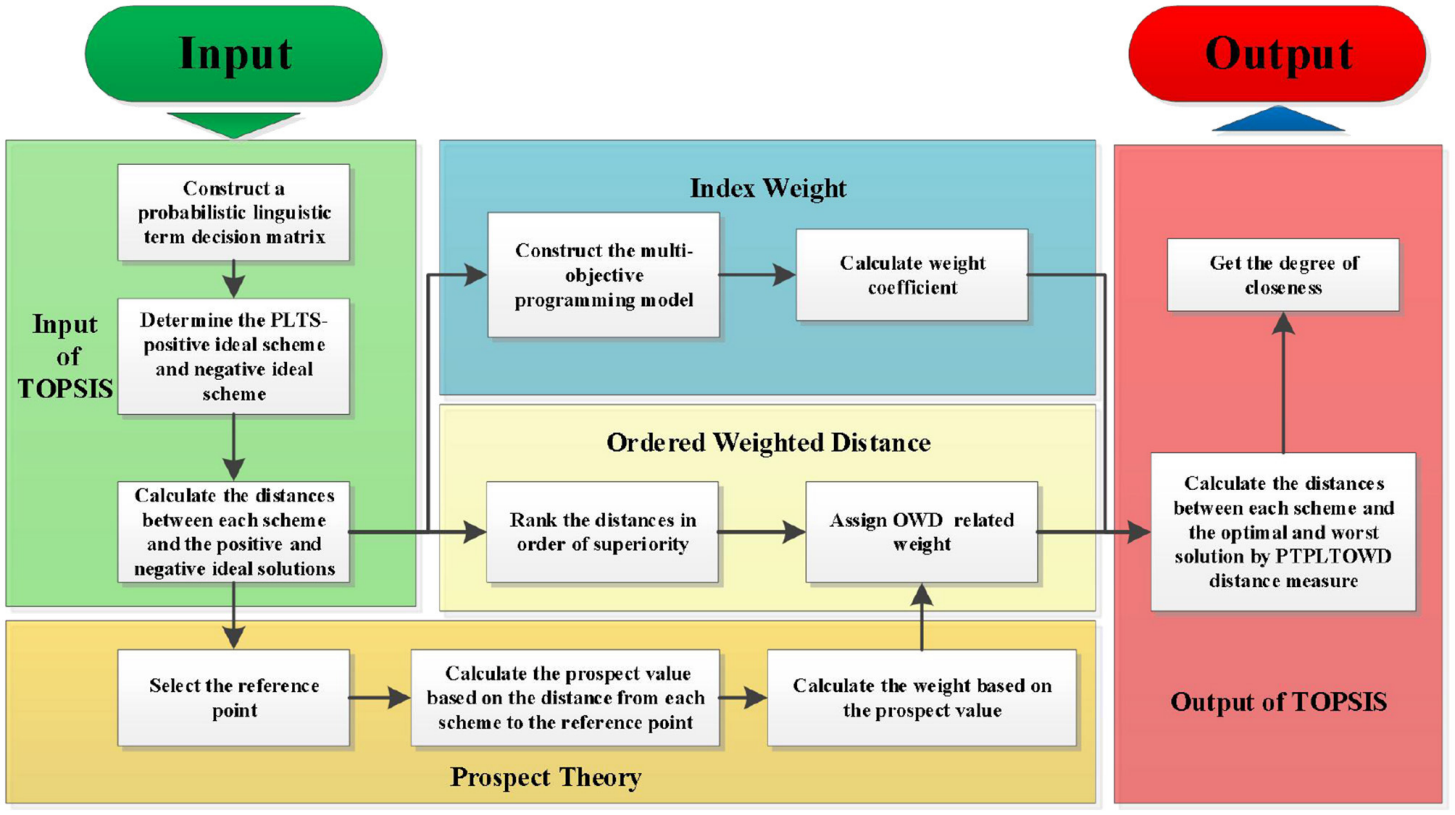
Flowchart of the PTPLTOWD-TOPSIS method.

Based on the above-mentioned information, then the main steps referring to the PTPLTOWD-TOPSIS method can be described in Figure 2:

**Step 1:** Construct evaluation matrix *R* = [*P*_*ij*_]_*m*×*n*_ according to the experts' assessment, where *P*_*ij*_ is the decision result given by the decision-maker in evaluating the attribute *c*_*j*_ ∈ *C* regarding the alternative *A*_*i*_ ∈ *A*. $P_{ij}=\left\{{L_{ij}}^{\left(k\right)}\left({p_{ij}}^{\left(k\right)}\right)|{L_{ij}}^{\left(k\right)}\;\in S,{p_{ij}}^{\left(k\right)}\geq 0,k=1,2,\cdots \;,\#L\left(p_{ij}\right)\right\}$, where *L*^(*k*)^ represents *k*, the linguistic term in *P*_*ij*_, ${p_{ij}}^{\left(k\right)}$ represents its corresponding probability, and *#L*(*p*_*ij*_) means the number of linguistic terms in *P*_*ij*_. In addition, we suppose the decision-makers are neutral in this study.

**Step 2:** Determine the PLTS-positive ideal scheme $L_{{}^{*}}$ and the PLTSs negative ideal scheme *L*_#_, which are determined as follows:


$$\begin{array}{l} L_{{}^{*}}=\left\{\langle c_{1},P_{{}^{*}1}\rangle ,\langle c_{2},P_{{}^{*}2}\rangle ,\cdots \;,\langle c_{n},P_{{}^{*}n}\rangle \right\} \end{array}$$

$$\begin{array}{l} L_{\#}=\left\{\langle c_{1},P_{\#1}\rangle ,\langle c_{2},P_{\#2}\rangle ,\cdots \;,\langle c_{n},P_{\#n}\rangle \right\}\; \end{array}$$

where $P_{{}^{*}j}$ and *P*_*#j*_ are defined as follows:


(14) $$\begin{array}{l} P_{{}^{*}j}=\{\begin{array}{c} \text{max }P_{ij},c_{j}\text{ is a benefit attribute},\\ \text{min }P_{ij},c_{j}\text{ is a cost attribute}, \end{array} \end{array}$$

(15) $$\begin{array}{l} P_{\#j}=\{\begin{array}{c} \text{min }P_{ij},c_{j\;}\text{is a benefit attribute},\\ \text{max }P_{ij},c_{j}\text{ is a cost attribute}, \end{array} \end{array}$$

Again, all indexes have been converted into benefit indexes before calculation, which means $P_{{}^{*}j}=\text{max }P_{ij}$ and *P*_*#j*_ = min *P*_*ij*_.

**Step 3:** Calculate the distances $d\left(P_{ij},P_{{}^{*}j}\right)$ and *d* (*P*_*ij*_, *P*_*#j*_) between each scheme *a*_*i*_ and the positive and negative ideal solutions.

**Step 4:** Calculate the index weights according to Equation (12).

**Step 5:** Select the reference point. For neutral decision-makers, the reference points in the case of cost- and benefit-based distance measures are $D^{Z}=\left(\frac{1}{n}\sum_{j=1}^{n}d\left(P_{ij},P_{{}^{*}j}\right),\frac{1}{n}\sum_{j=1}^{n}d\left(P_{ij},P_{\#j}\right)\right)$, respectively.

**Step 6:** Calculate the distance Δx = (*dx*^+^, *dx*^−^) between each scheme and the reference points according to the above reference points.

**Step 7:** According to Equation (8), the prospect value is calculated based on Δx:


(16) $$\begin{array}{l} V_{j}=\{\begin{array}{c} {\left(\Delta x\right)}^{0.88},\;\Delta x\geq 0\\ -2.25{\left(-\Delta x\right)}^{0.88},\;\Delta x<0 \end{array} \end{array}$$

To ensure that the prospect value is positive, we make the following transformation to the prospect value: $V_{j}^{\prime }=\text{max }\left\{V_{1},V_{2},\cdots \;,V_{n}\right\}-\text{min }\left\{V_{1},V_{2},\cdots \;,V_{n}\right\}+V_{j}$. After that, $V_{j}^{\prime }$ is still denoted as *V*_*j*_.

**Step 8:** According to Equation (11), the prospect value *V*_*j*_ is used to calculate the corresponding weight.

**Step 9:** The obtained weights from the above step are assigned to the distances $d\;\left(P_{ij},P_{{}^{*}j}\right)$ and s*d* (*P*_*ij*_, *P*_*#j*_) from each scheme to the optimal and worst solutions in order from the largest to smallest.

**Step 10:** Calculate the weight associated with the distance measure based on Step 6, then obtain the $PTPLTOWD^{\prime }\;\left(A_{i},A^{-}\right)$ and $PTPLTOWD^{\prime }\;\left(A_{i},A^{+}\right)$ between each alternative and the positive and negative ideal solutions according to Equation (13). (In general, λ equals 2. Other cases are discussed later in this article.)


(17) $$\begin{array}{l} PTPLTOWD^{\prime }\;(A_{i},A^{+})={\left(\overset{n}{\underset{j=1}{\sum }}\varphi_{j}{\;(\omega_{j}d_{PLT}\;({A_{i}}_{\sigma \left(j\right)},{A^{+}}_{\sigma \left(j\right)}))}^{2}\right)}^{\frac{1}{2}} \end{array}$$

(18) $$\begin{array}{l} PTPLTOWD^{\prime }\;\left(A_{i},A^{-}\right)={\left(\overset{n}{\underset{j=1}{\sum }}\varphi_{j}\;{\left(\omega_{j}d_{PLT}\;({A_{i}}_{\sigma \left(j\right)},{A^{-}}_{\sigma \left(j\right)})\right)}^{2}\right)}^{\frac{1}{2}} \end{array}$$

**Step 11**: Calculate the relative closeness based on PTPLTOWD.


(19) $$\begin{array}{l} R_{i}=\frac{PTPLTOWD^{\prime }\;\left(A_{i},A^{-}\right)}{PTPLTOWD^{\prime }\;\left(A_{i},A^{-}\right)+PTPLTOWD^{\prime }\;\left(A_{i},A^{+}\right)}. \end{array}$$

Among them, *R*_*i*_ is the relative closeness of each alternative, and the larger the value, the closer the scheme *A*_*i*_ is to the positive ideal solution and the farther it is from the negative ideal solution.

## 5. Case study

In this section, we explore the application of the innovative index system and the proposed PTPLTOWD-TOPSIS method in the evaluation of the common prosperity level of cities in the Zhejiang province.

### 5.1. Case calculation analysis

On 19 July 2021, the “Zhejiang High-quality Development and Construction of Common Prosperity Demonstration Zone Implementation Plan (2021–2025)” was officially released, which proposes to widen the effective path of the “getting rich first” strategy by promoting the coordinated development of rural and urban areas as a first demonstration. This strategy of the central government was designed to achieve a mutually beneficial, balanced development solution. Zhejiang province needs to achieve institutional reforms to share the fruits of economic development.

“Between different regions in Zhejiang province, between big cities and small cities, and between cities and rural areas, it is vital to promote the reform of public services and social security by following the process of urbanization.” By 2021, Zhejiang province was destined to become a model for common prosperity in China. Zhejiang province has a solid foundation in terms of economy, society, and the rule of law, and the awareness of reform and innovation is strong in various regions. Its construction space and potential are significant. Therefore, to demonstrate the practicability and effectiveness of the above methods, we conducted an empirical test on the common prosperity practice in Zhejiang province.

This example considers 11 prefecture-level cities in Zhejiang province, including Hangzhou, Ningbo, Jiaxing, Huzhou, Wenzhou, Jinhua, Quzhou, Zhoushan, Shaoxing, Taizhou, and Lishui, for evaluation, named *A*_1_−*A*_11_, respectively. Factors are named as follows: *c*_1_—income gap, *c*_2_— economic level, *c*_3_—culture and recreation, *c*_4_—infrastructure, *c*_5_—urbanization rate, *c*_6_—life expectancy, and *c*_7_—employment rate.

Each expert in the evaluation team used seven-value language terms to evaluate each index attribute—using seven-level criteria: *extremely poor, poor, relatively poor, average, good, better*, and *excellent* for independent evaluation. For convenience, the seven-valued language term set is denoted as *S* = (*s*_0_, *s*_1_, *s*_2_, *s*_3_, *s*_4_, *s*_5_, *s*_6_). Based on the language evaluation of each expert on each attribute index of each evaluation object, the probabilistic linguistic evaluation matrix is obtained, as shown in Table 1.

Table 1Probabilistic linguistic evaluation matrix.

**Table:** 

	***C*_1_**	***C*_2_**	***C*_3_**	***C*_4_**
*A*_1_	{*s*_1_ (0.5), *s*_2_ (0.3), *s*_3_ (0.2)}	{*s*_5_ (0.2), *s*_6_ (0.8)}	{*s*_4_ (0.1), *s*_5_ (0.1), *s*_6_ (0.8)}	{*s*_4_ (0.1), *s*_5_ (0.2), *s*_6_ (0.7)}
*A*_2_	{*s*_0_ (0.4), *s*_1_ (0.3), *s*_2_ (0.2), *s*_3_ (0.1)}	{*s*_5_ (0.3), *s*_6_ (0.7)}	{*s*_4_ (0.3), *s*_5_ (0.3), *s*_6_ (0.4)}	{*s*_4_ (0.2), *s*_5_ (0.5), *s*_6_ (0.3)}
*A*_3_	{*s*_0_ (0.6), *s*_1_ (0.4)}	{*s*_4_ (0.2), *s*_5_ (0.6), *s*_6_ (0.2)}	{*s*_3_ (0.1), *s*_4_ (0.1), *s*_5_ (0.6), *s*_6_ (0.2)}	{*s*_4_ (0.7), *s*_5_ (0.3)}
*A*_4_	{*s*_2_ (0.6), *s*_3_ (0.2), *s*_4_ (0.2)}	{*s*_3_ (0.2), *s*_4_ (0.5), *s*_5_ (0.3)}	{*s*_4_ (0.6), *s*_5_ (0.4)}	{*s*_2_ (0.3), *s*_3_ (0.4), *s*_4_ (0.3)}
*A*_5_	{*s*_2_ (0.3), *s*_3_ (0.3), *s*_4_ (0.4)}	{*s*_2_ (0.3), *s*_3_ (0.5), *s*_4_ (0.2)}	{*s*_3_ (0.1), *s*_4_ (0.7), *s*_5_ (0.2)}	{*s*_3_ (0.2), *s*_4_ (0.8)}
*A*_6_	{*s*_3_ (0.1), *s*_4_ (0.8), *s*_5_ (0.1)}	{*s*_3_ (0.3), *s*_4_ (0.7)}	{*s*_3_ (0.2), *s*_4_ (0.3), *s*_5_ (0.4), *s*_6_ (0.1)}	{*s*_3_ (0.3), *s*_4_ (0.6), *s*_5_ (0.1)}
*A*_7_	{*s*_2_ (0.2), *s*_3_ (0.3), *s*_4_ (0.5)}	{*s*_1_ (0.1), *s*_2_ (0.3), *s*_3_ (0.4), *s*_4_ (0.2)}	{*s*_2_ (0.2), *s*_3_ (0.6), *s*_4_ (0.2)}	{*s*_1_ (0.4), *s*_2_ (0.5), *s*_3_ (0.1)}
*A*_8_	{*s*_4_ (0.1), *s*_5_ (0.8), *s*_6_ (0.1)}	{*s*_0_ (0.5), *s*_1_ (0.5)}	{*s*_1_ (0.1), *s*_2_ (0.5), *s*_3_ (0.4)}	{*s*_0_ (0.4), *s*_1_ (0.3), *s*_2_ (0.2), *s*_3_ (0.1)}
*A*_9_	{*s*_4_ (0.6), *s*_5_ (0.4)}	{*s*_0_ (0.4), *s*_1_ (0.4), *s*_2_ (0.2)}	{*s*_0_ (0.3), *s*_1_ (0.7)}	{*s*_0_ (0.3), *s*_1_ (0.5), *s*_2_ (0.2)}
*A*_10_	{*s*_2_ (0.3), *s*_3_ (0.7)}	{*s*_0_ (0.2), *s*_1_ (0.5), *s*_2_ (0.3)}	{*s*_2_ (0.2), *s*_3_ (0.5), *s*_4_ (0.3)}	{*s*_2_ (0.1), *s*_3_ (0.6), *s*_4_ (0.2), *s*_5_ (0.1)}
*A*_11_	{*s*_3_ (0.2), *s*_4_ (0.3), *s*_5_ (0.4), *s*_6_ (0.1)}	{*s*_0_ (0.7), *s*_1_ (0.2), *s*_2_ (0.1)}	{*s*_1_ (0.6), *s*_2_ (0.4)}	{*s*_0_ (0.5), *s*_1_ (0.3), *s*_2_ (0.2)}

**Table:** 

	*C*_5_	*C*_6_	*C*_7_
*A*_1_	{*s*_4_ (0.1), *s*_5_ (0.6), *s*_6_ (0.3)}	{*s*_4_ (0.3), *s*_5_ (0.5), *s*_6_ (0.2)}	{*s*_3_ (0.2), *s*_4_ (0.3), *s*_5_ (0.5)}
*A*_2_	{*s*_4_ (0.1), *s*_5_ (0.6), *s*_6_ (0.3)}	{*s*_3_ (0.1), *s*_4_ (0.6), *s*_5_ (0.3)}	{*s*_4_ (0.3), *s*_5_ (0.3), *s*_6_ (0.4)}
*A*_3_	{*s*_3_ (0.1), *s*_4_ (0.5), *s*_5_ (0.4)}	{*s*_3_ (0.3), *s*_4_ (0.7)}	{*s*_3_ (0.2), *s*_4_ (0.3), *s*_5_ (0.5)}
*A*_4_	{*s*_3_ (0.4), *s*_4_ (0.3), *s*_5_ (0.2), *s*_6_ (0.1)}	{*s*_3_ (0.1), *s*_4_ (0.2), *s*_5_ (0.6), *s*_6_ (0.1)}	{*s*_3_ (0.3), *s*_4_ (0.6), *s*_5_ (0.1)}
*A*_5_	{*s*_3_ (0.2), *s*_4_ (0.7), *s*_5_ (0.1)}	{*s*_3_ (0.2), *s*_4_ (0.2), *s*_5_ (0.6)}	{*s*_2_ (0.3), *s*_3_ (0.5), *s*_4_ (0.2)}
*A*_6_	{*s*_3_ (0.4), *s*_4_ (0.6)}	{*s*_3_ (0.1), *s*_4_ (0.3), *s*_5_ (0.5), *s*_6_ (0.1)}	{*s*_2_ (0.2), *s*_3_ (0.6), *s*_4_ (0.2)}
*A*_7_	{*s*_1_ (0.2), *s*_2_ (0.5), *s*_3_ (0.2), *s*_4_ (0.1)}	{*s*_2_ (0.3), *s*_3_ (0.4), *s*_4_ (0.3)}	{*s*_2_ (0.3), *s*_3_ (0.7)}
*A*_8_	{*s*_0_ (0.6), *s*_1_ (0.2), *s*_2_ (0.2)}	{*s*_3_ (0.1), *s*_4_ (0.6), *s*_5_ (0.3)}	{*s*_0_ (0.4), *s*_1_ (0.3), *s*_2_ (0.3)}
*A*_9_	{*s*_0_ (0.2), *s*_1_ (0.5), *s*_2_ (0.2), *s*_3_ (0.1)}	{*s*_2_ (0.2), *s*_3_ (0.5), *s*_4_ (0.2), *s*_5_ (0.1)}	{*s*_0_ (0.2), *s*_1_ (0.3), *s*_2_ (0.5)}
*A*_10_	{*s*_1_ (0.2), *s*_2_ (0.6), *s*_3_ (0.2)}	{*s*_2_ (0.2), *s*_3_ (0.3), *s*_4_ (0.3), *s*_5_ (0.2)}	{*s*_1_ (0.3), *s*_2_ (0.6), *s*_3_ (0.1)}
*A*_11_	{*s*_0_ (0.5), *s*_1_ (0.3), *s*_2_ (0.2)}	{*s*_1_ (0.2), *s*_2_ (0.3), *s*_3_ (0.4), *s*_4_ (0.1)}	{*s*_0_ (0.6), *s*_1_ (0.4)}

The specific analysis of the PTLTOWD-TOPSIS method proposed in this study and the corresponding evaluation results are shown as follows.

**Step 1:** Construct the probabilistic linguistic evaluation matrix *R* = [*P*_*ij*_]_*m*×*n*_ based on the decision information. Details are presented in Table 1.

**Step 2:** Determine the PLTS-positive ideal scheme $L_{{}^{*}}$ and PLTS-negative ideal scheme *L*_#_:


$$\begin{array}{l} L_{{}^{*}}=\left(\begin{array}{l} \langle c_{1},\left\{{s_{4}\;(0.1),s}_{5}\;(0.8),s_{6}\;(0.1)\right\}\rangle ,\langle c_{2},\left\{s_{5}\;(0.2),s_{6}\;(0.8)\right\}\rangle ,\\ \;\langle c_{3},\left\{{s_{4}\;(0.1),s}_{5}\;(0.1),s_{6}\;(0.8)\right\}\rangle ,\\ \;\langle c_{4},\left\{{s_{4}\;(0.1),s}_{5}\;(0.2),s_{6}\;(0.7)\right\}\rangle ,\\ \;\langle c_{5},\left\{{s_{4}\;(0.1),s}_{5}\;(0.6),s_{6}\;(0.3)\right\}\rangle ,\\ \;\langle c_{6},\left\{{s_{4}\;(0.3),s}_{5}\;(0.5),s_{6}\;(0.2)\right\}\rangle ,\\ \;\langle c_{7},\left\{{s_{4}\;(0.2),s}_{5}\;(0.3),s_{6}\;(0.5)\right\}\rangle \end{array}\right) \end{array}$$

$$\begin{array}{l} L_{\#}=\left(\begin{array}{l} \langle c_{1},\left\{s_{0}\;(0.6),s_{1}\;(0.4)\right\}\rangle ,\langle c_{2},\left\{s_{0}\;(0.7),s_{1}\;(0.2),s_{2}\;(0.1)\right\}\rangle ,\\ \;\langle c_{3},\left\{s_{0}\;(0.3),s_{1}\;(0.7)\right\}\rangle ,\\ \;\langle c_{4},\left\{s_{0}\;(0.5),s_{1}\;(0.3),s_{2}\;(0.2)\right\}\rangle ,\\ \;\langle c_{5},\left\{s_{0}\;(0.6),s_{1}\;(0.2),s_{2}\;(0.2)\right\}\rangle ,\\ \langle c_{6},\left\{s_{1}\;(0.2),s_{2}\;(0.3),s_{3}\;(0.4),s_{4}\;(0.1)\right\}\rangle ,\\ \;\langle c_{7},\left\{s_{0}\;(0.6),s_{1}\;(0.4)\right\}\rangle \end{array}\right)\; \end{array}$$

**Step 3:** Calculate the distances $d\;\left(P_{ij},P_{{}^{*}j}\right)$ and *d* (*P*_*ij*_, *P*_*#j*_) between each scheme *A*_*i*_ and the positive and negative ideal solutions.

**Step 4:** Obtain the index weights according to Equation (12):


$$\begin{array}{l} \omega =\;\left[0.045,0.089,0.262,0.182,0.001,0.356,0.065\right]. \end{array}$$

**Step 5:** Select the reference point. For neutral decision-makers, the reference points in the case of cost- and benefit-based distance measures are $D^{Z}=\left(\frac{1}{n}\sum_{j=1}^{n}d_{ij}^{+},\frac{1}{n}\sum_{j=1}^{n}d_{ij}^{-}\right)$, respectively. The results are presented in Table 2.

**Table 2:** Reference points of cities.

***D* ^*Z*^**	***A*_1_**	***A*_2_**	***A*_3_**	***A*_4_**	***A*_5_**	***A*_6_**	***A*_7_**	***A*_8_**	***A*_9_**	***A*_10_**	***A*_11_**
$\frac{1}{n}\sum_{j=1}^{n}d_{ij}^{+}$	1.67	2.9	5.17	6.33	7.03	6.71	7.31	5.23	6.09	6.86	5.87
$\frac{1}{n}\sum_{j=1}^{n}d_{ij}^{-}$	5.31	5	4.3	4.29	4.04	4.47	2.36	2.13	1.24	2.39	0.83

**Step 6:** Calculate the distance Δx = (*dx*^+^, *dx*^−^) between each scheme and its corresponding reference point $\left(\frac{1}{n}\sum_{j=1}^{n}d_{ij}^{+},\frac{1}{n}\sum_{j=1}^{n}d_{ij}^{-}\right)$. It is worth mentioning that the reference points of each city are obtained by the distance between its probabilistic language and the optimal solution and the worst solution, respectively. Hence, its reference points are also a pair. $\frac{1}{n}\sum_{j=1}^{n}d_{ij}^{+}$ is the reference point obtained by the distance from the optimal solution, and $\frac{1}{n}\sum_{j=1}^{n}d_{ij}^{-}$ is the reference point obtained by the deviation from the worst solution.

**Step 7:** According to the obtained Δx, *V*_*ij*_ is calculated by using Equations (9) and (16). For the reference points that are paired, the resulting prospect value is also paired. The prospect value $V_{ij}^{{}^{*}}$ given by the reference point $\frac{1}{n}\sum_{j=1}^{n}d_{ij}^{+}$ is called the prospect value of the optimal reference point, while the prospect value $V_{ij}^{\#}$ given by the reference point $\frac{1}{n}\sum_{j=1}^{n}d_{ij}^{-}$ is called the prospect value of the worst reference point.

**Step 8:** According to the two groups of prospect values obtained above, the weight of prospect theory is calculated, respectively, as shown in Tables 3, 4. Among them, $\omega_{{}^{*}}$ is the prospect theory weight obtained from the prospect value of the optimal reference point, which is called the prospect theory weight of the optimal reference point; ω_#_ is the prospect theory weight obtained from the prospect value of the worst reference point, which is called the prospect theory weight of the worst reference point.

**Table 3:** Prospect theory weight of optimal reference point $\omega_{{}^{*}}$.

	***C*_1_**	***C*_2_**	***C*_3_**	***C*_4_**	***C*_5_**	***C*_6_**	***C*_7_**
*A*_1_	0.0863	0.1676	0.1604	0.1625	0.1499	0.1320	0.1413
*A*_2_	0.0533	0.1758	0.1635	0.1667	0.1567	0.1304	0.1536
*A*_3_	0.0006	0.1966	0.1834	0.1690	0.1542	0.1134	0.1826
*A*_4_	0.0354	0.1876	0.1850	0.1417	0.1562	0.1491	0.1450
*A*_5_	0.0369	0.1573	0.1924	0.1866	0.1650	0.1455	0.1163
*A*_6_	0.0761	0.1736	0.1857	0.1692	0.1397	0.1425	0.1131
*A*_7_	0.0527	0.2167	0.2096	0.1353	0.1269	0.1138	0.1450
*A*_8_	0.1920	0.1188	0.1733	0.1291	0.0904	0.1993	0.0971
*A*_9_	0.1840	0.1451	0.1165	0.1377	0.1196	0.1789	0.1182
*A*_10_	0.0374	0.1229	0.2151	0.2374	0.1251	0.1559	0.1063
*A*_11_	0.2045	0.1394	0.1734	0.1432	0.1073	0.1494	0.0828

**Table 4:** Prospect theory weight of the worst reference point ω_#_.

	***C*_1_**	***C*_2_**	***C*_3_**	***C*_4_**	***C*_5_**	***C*_6_**	***C*_7_**
*A*_1_	0.1633	0.2072	0.1094	0.1785	0.1396	0.1191	0.0827
*A*_2_	0.1278	0.2051	0.0913	0.1611	0.1565	0.0792	0.1790
*A*_3_	0.1265	0.1924	0.1055	0.1460	0.1374	0.0904	0.2019
*A*_4_	0.2984	0.1540	0.0924	0.0387	0.1158	0.1763	0.1244
*A*_5_	0.3516	0.0681	0.0880	0.1268	0.1277	0.1707	0.0670
*A*_6_	0.4265	0.1073	0.0823	0.0961	0.0680	0.1751	0.0448
*A*_7_	0.3519	0.1384	0.0827	0.0658	0.0928	0.1338	0.1345
*A*_8_	0.5330	0.0295	0.0915	0.0495	0.0098	0.2277	0.0590
*A*_9_	0.4037	0.0928	0.0223	0.0857	0.1013	0.1668	0.1274
*A*_10_	0.3679	0.0460	0.0933	0.1550	0.0845	0.1614	0.0918
*A*_11_	0.4001	0.0907	0.0777	0.0978	0.0914	0.1523	0.0900

**Step 9:** $d\;\left(P_{ij},P_{{}^{*}j}\right)$ and *d* (*P*_*ij*_, *P*_*#j*_)) are aggregated and ranked in order of largest to smallest.

**Step 10:** According to Equations (17) and (18), calculate distances $PTPLTOWD^{\prime }\;\left(A_{i},A^{-}\right)$ and $PTPLTOWD^{\prime }\;\left(A_{i},A^{+}\right)$ between each scheme and the positive and negative ideal alternatives.

**Step 11:** Compute the relative closeness based on PTPLTOWD and Equation (19), which is shown in Table 5.

**Table 5:** The relative closeness of alternatives.

	**City**	***R*_*i*_**
*A*_1_	Hangzhou	0.9
*A*_2_	Ningbo	0.67
*A*_3_	Wenzhou	0.5
*A*_4_	Jiaxing	0.57
*A*_5_	Huzhou	0.49
*A*_6_	Shaoxing	0.55
*A*_7_	Jinhua	0.42
*A*_8_	Quzhou	0.42
*A*_9_	Zhoushan	0.23
*A*_10_	Taizhou	0.43
*A*_11_	Lishui	0.19

It can be observed that the development of common prosperity in the Zhejiang province—Hangzhou, Ningbo, Jiaxing, Shaoxing, Wenzhou, Huzhou, Taizhou, Quzhou, Jinhua, Zhoushan, and Lishui—ranges from high to relatively low.

### 5.2. Comparative analysis

To better illustrate the advantages of the method proposed in this study, we select the PLTOWD-TOPSIS and PLT-TOPSIS methods to compare and analyze the above examples. By comparing the closeness of 11 cities presented by the current two evaluation models, we illustrate the advantages and effectiveness of introducing the prospect theory in PTPLTOWD-TOPSIS proposed in this study.

**Figure 3:**
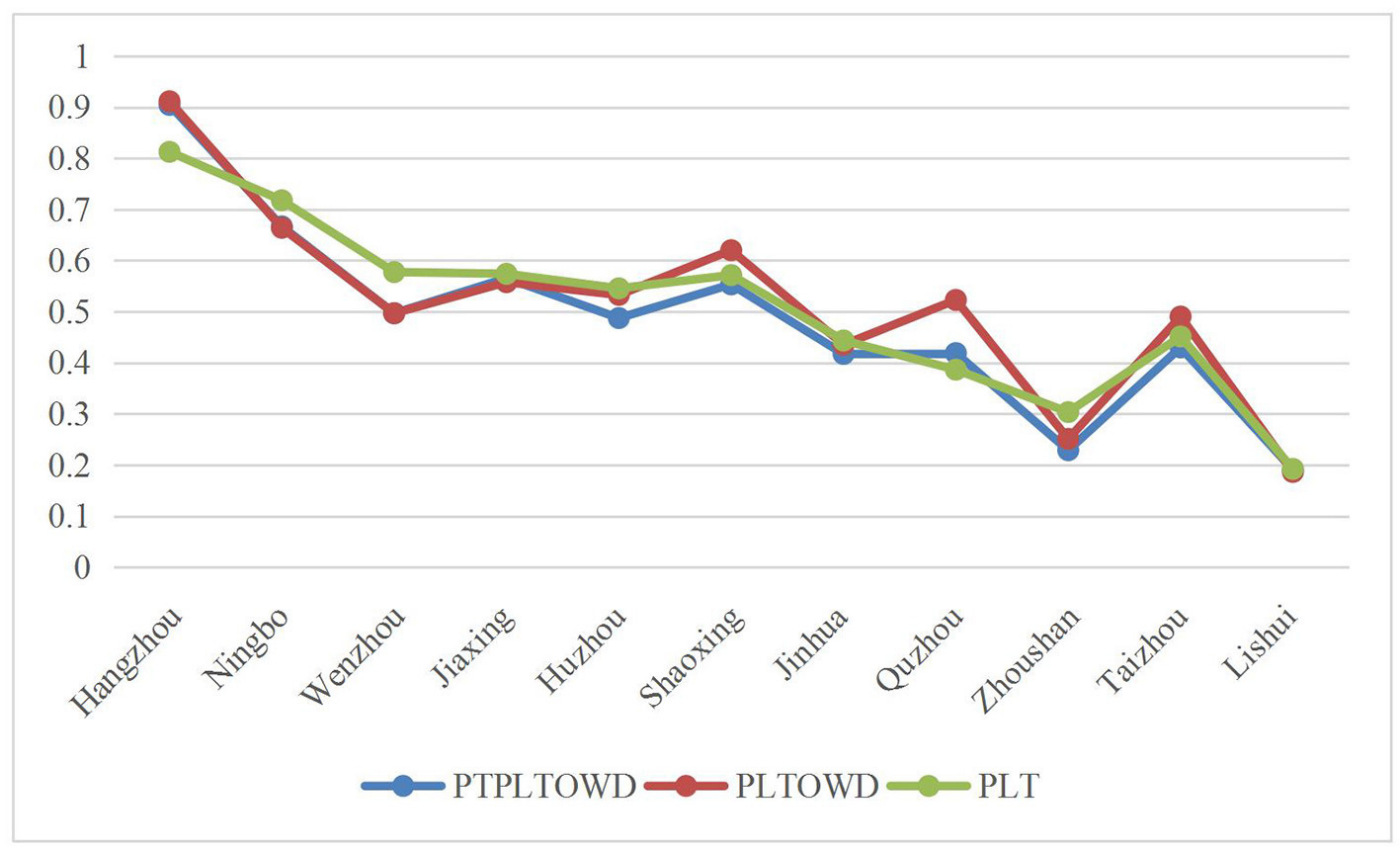
Degree of closeness under different methods.

To illustrate the advantages of the proposed approach, we first employ the general PLTOWD measure as a comparison. It is worth mentioning that the weight of the PLTOWD method here adopts the widely used normal distribution method (Xu and Chen, 2008), while the associated weights with the PTPLTOWD measure in this study are determined by the prospect theory. It can be found from Figure 3 that the two results of the first four cities, Hangzhou, Ningbo, Wenzhou, and Jiaxing, are relatively similar, while the two results of the next seven cities gradually show differences, among which, Quzhou has the largest difference. The main reason for this difference is that prospect theory reflects the preferences of decision-makers. At the same time, we can also find that cities that perform well under prospect theory perform well on some indicators. As for the pure PLT operator, it is observed that it simply integrates the evaluation values of the indicators and does not reflect the gap between the aggregation results of the scheme and the positive and negative ideal alternatives.

Overall, by considering prospect theory based on PLTOWD, the proposed PTPLTOWD measure accounts for the psychology of decision-makers and is more responsive. It improves the subjective value or feelings of decision-makers when they face losses and gains in the actual decision-making process so that the conclusions are more scientific and reasonable.

### 5.3. Analysis of sensitivity

**Figure 4:**
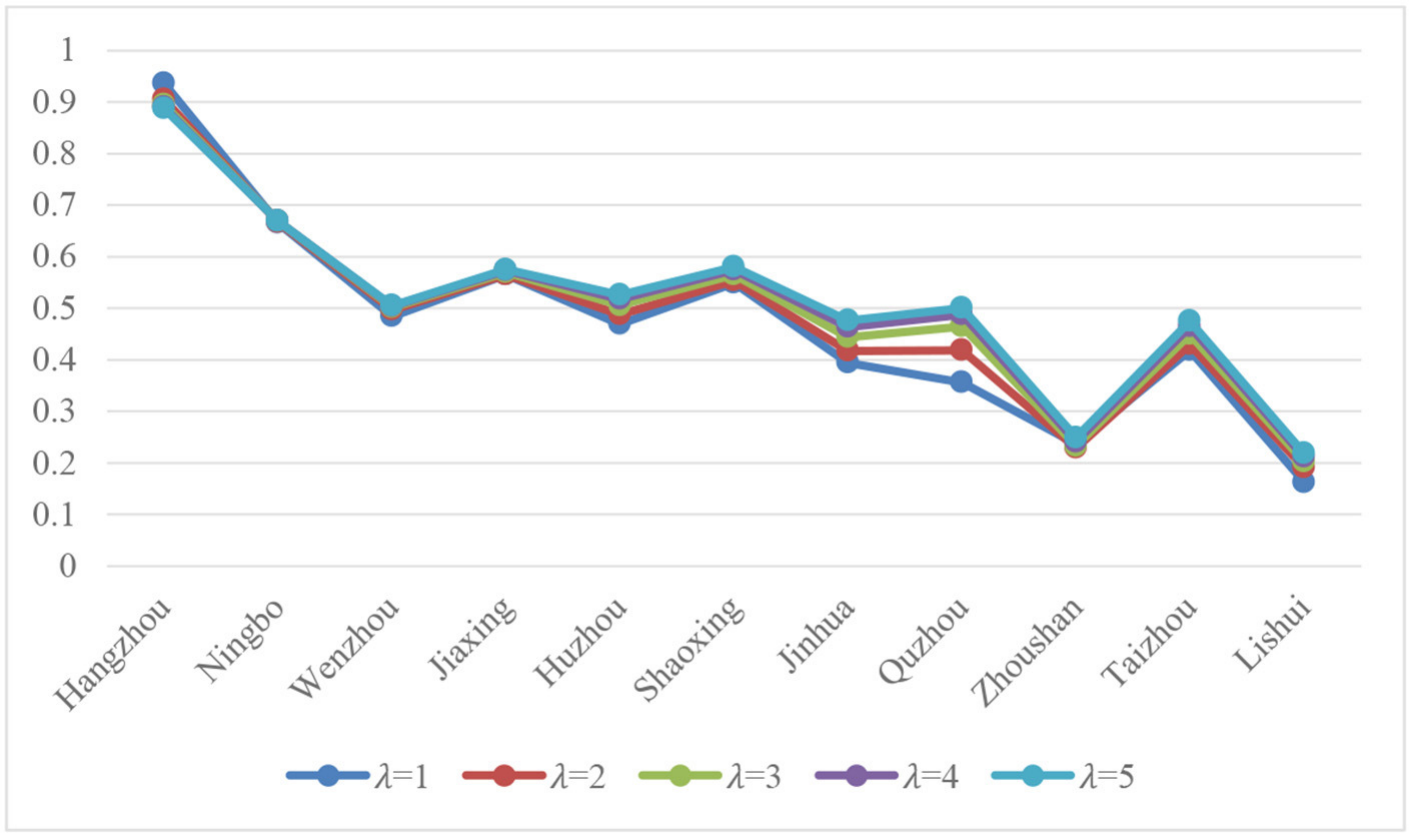
Influence of λ on the degree of closeness.

In this part, we explore the influence of the PTPLTOWD measure on the result by changing λ, which is shown in Figure 4.

We can find that the results of several curves are relatively similar and have nothing to do with the value of λ, which shows that the methods proposed in this study have good stability. Of course, the results may be slightly different in specific cities such as Quzhou, but this does not affect the overall results, and λ is generally taken as 2 when making decisions. In the context of this study, the result with λ = 2 is satisfactory.

## 6. Conclusion

On the background of the commonwealth level of the people, aiming to provide an academic reference for related fields, this study introduces the prospect theory into the fuzzy evaluation field under complex PLTS situations. The main innovation advantages of this study are as follows: (1) We proposed a PTPLTOWD measure based on prospect theory that is an extension of the PLTOWD measure and able to better reflect the preferences of decision-makers; (2) we extended the traditional TOPSIS method based on prospect theory and ordered the weighted measure to construct a novel PTPLTOWD-TOPSIS evaluation framework, providing a simpler and more efficient probabilistic linguistic decision-making method; (3) we constructed a set of scientific evaluation index systems for the common prosperity level by reading literature; and (4) we applied the presented method to the evaluation of the common prosperity level of cities in Zhejiang province and obtained a scientific evaluation result. The results are compared with the previous method, and the effect is remarkable. This demonstrates the practicability and effectiveness of the PTPLTOWD-TOPSIS approach. The evaluation of this study has a certain reference significance in reality.

The findings show that the level of common prosperity of the 11 prefecture-level cities in Zhejiang province, ranked from high to relatively low, is Hangzhou, Ningbo, Jiaxing, Shaoxing, Wenzhou, Huzhou, Taizhou, Quzhou, Jinhua, Zhoushan, and Lishui. Cities should make targeted improvements based on the shortcomings currently reflected to achieve common prosperity. Furthermore, it is observed that the PTPLOWD has a broad application prospect. In the future, it will be considered for application in other fuzzy evaluation fields. In addition to prospect theory, regret theory can also be applied in the PTOWD and field of fuzzy evaluation.

There are two methodological topics that need to be highlighted. First, the proposed evaluation system contains seven indicators, which are relatively simple and easy to operate during calculation. We can further add or expand some other related indicators in the subsequent to make them more scientific and comprehensive, which may be a potential area for future research. Second, determining how the importance of an index is a classic issue in multi-attribute evaluation problems. Different weights should be assigned to different indicators according to actual situations. In this case, we have utilized a multi-objective programming method to determine the objective weights of indexes. Subjective methods by considering the people's subjective judgment, such as the social network trust model (Zeng et al., 2022) and BWM method (Zhang N. et al., 2022), can be considered regarding the allocation of weights in practical evaluation.

## Data availability statement

The raw data supporting the conclusions of this article will be made available by the authors, without undue reservation.

## Author contributions

Writing—original draft preparation: FY and TJ. Writing—review and editing: EZ and SZ. Methodology: EZ and DW. Supervision: SZ and DW. All authors have read and agreed to the published version of the manuscript.
